# Changes in epidemic methicillin-resistant *Staphylococcus aureus* strains in a Japanese hospital in Tokyo, Japan

**DOI:** 10.1016/j.ijregi.2026.101024

**Published:** 2026-09-29

**Authors:** Takuma Yoshida, Rika Sawara, Yuma Tomida, Mayu Sato, Yuki Okura, Hidetoshi Masuda, Taiga Natori, Takemi Ozeki, Tatsuya Numadate, Natsuki Oda, Megumi Sakaihori, Rika Watanabe, Hinako Kawasaki, Natsuki Fukuda, Tomomi Akasu, Shunsuke Takadama, Hiroshi Kaneko, Norihisa Noguchi, Yuji Hirai, Hidemasa Nakaminami

**Affiliations:** 1 Department of Clinical Microbiology, School of Pharmacy, Tokyo University of Pharmacy and Life Sciences, Hachioji, Tokyo, Japan; 2 Department of Infectious Diseases, Tokyo Medical University Hachioji Medical Centre, Hachioji, Tokyo, Japan

**Keywords:** Japanese hospital, Methicillin-resistant *Staphylococcus aureus*, Staphylococcal cassette chromosome *mec* type IV, USA400/J

## Abstract

**Objectives:**

In recent years, methicillin-resistant *Staphylococcus aureus* (MRSA) isolates from Japanese hospitals have predominantly comprised community-acquired MRSA (CA-MRSA) carrying staphylococcal cassette chromosome *mec* (SCC*mec*) type IV in most regions. Certain CA-MRSA strains produce toxins, notably, Panton–Valentine leukocidin (PVL), a leukocyte-lytic toxin, and may cause infections in otherwise healthy individuals. This study aimed to clarify the current epidemiologic status of MRSA in a Japanese hospital.

**Methods:**

In total, 1479 MRSA isolates collected from a Japanese university hospital between 2016 and 2023 were analyzed. SCC*mec* types and various genes were detected using polymerase chain reaction. Molecular epidemiologic analyses were performed using multilocus sequence typing and pulsed-field gel electrophoresis. Antimicrobial susceptibility was tested using the agar dilution method.

**Results:**

The proportion of SCC*mec* type IV MRSA cases increased from 45.9% to 86.4% over 8 years. The PVL-positive MRSA isolation rate ranged from 6.4% to 15.9%. PVL-negative strains predominated throughout the study period. Among the PVL-negative SCC*mec* type IV strains, the USA400/J clone belonging to sequence type 1 accounted for 40.6% of the isolates, whereas the sequence type 8 CA-MRSA/J clone carrying SCC*mec* type IVl accounted for 15.1%. The USA400/J clone exhibited high resistance to levofloxacin but remained susceptible to clindamycin and minocycline, displaying a characteristic antimicrobial susceptibility pattern.

**Conclusion:**

The USA400/J clone of CA-MRSA was predominant in the studied hospital. Strains with characteristics consistent with the USA400/J clone have also been reported in other regions of Japan, suggesting that this clone may be widely disseminated in Japan.

## Highlights

• Staphylococcal cassette chromosome *mec* type IV methicillin-resistant *Staphylococcus aureus* increased markedly from 45.9% to 86.4% between 2016 and 2023.
• The USA400/J clone predominated among Panton–Valentine leukocidin–-negative Staphylococcal cassette chromosome *mec* type IV methicillin-resistant *Staphylococcus aureus* strains in a Japanese hospital.
• The USA400/J clone showed a characteristic antimicrobial susceptibility profile.

## Introduction

*Staphylococcus aureus* is a commensal bacterium found on human skin, in the nasal cavity, and at other body sites. However, it is also a causative agent of a wide range of infections. Methicillin-resistant *S. aureus* (MRSA) is the most frequently isolated antimicrobial-resistant bacterium in hospitals and is considered a key pathogen requiring stringent infection control measures.

MRSA arises through the acquisition of the *mecA* gene, which encodes penicillin-binding protein 2′, with a low affinity for β-lactam antibiotics [1]. The *mecA* gene is located on the chromosome within a mobile genetic element called the staphylococcal cassette chromosome (SCC), designated as SCC*mec* [2]. SCC*mec* is composed of the *mec* gene and the *ccr* gene complex, which mediates the integration and excision of the SCC. Based on the combination of these elements, SCC*mec* is classified into types I-XV [3]. MRSA strains are broadly classified as community-acquired MRSA (CA-MRSA) and healthcare–associated MRSA (HA-MRSA), which generally differ in their SCC*mec* types and antimicrobial susceptibility profiles [4]. According to the 2024 Japanese guidelines, HA-MRSA is defined as having SCC*mec* types I, II, and III, whereas CA-MRSA corresponds to types IV and V [5].

HA-MRSA isolates from Japan are often multidrug-resistant and include strains producing toxic shock syndrome toxin-1 (TSST-1), encoded by *tst*. In contrast, CA-MRSA is generally susceptible to a range of antimicrobial agents other than β-lactams, and some strains produce Panton–Valentine leukocidin (PVL), a leukocyte-lytic toxin encoded by *lukS/F-PV* [6]. PVL-positive MRSA has been associated with severe infections, including skin and soft tissue infections and necrotizing pneumonia [7]. Before 2013, MRSA isolates from Japanese hospitals were predominantly clonal complex (CC) 5-SCC*mec* type II strains, primarily represented by sequence type (ST) 5 strains, which can be identified through multilocus sequence typing (MLST) and are collectively referred to as the New York/Japan clone [8]. Since 2014, however, ST1 strains, corresponding to CC1-SCC*mec* type IV strains, have accounted for approximately half of all isolates [9]. ST1-SCC*mec* type IV (ST1-IV) strains have been isolated worldwide. Among these clones, the PVL-positive USA400 lineage, originally defined using a pulsed-field gel electrophoresis (PFGE)-based typing scheme, is a well-characterized ST1-IV lineage [[10], [11], [12]]. In contrast, many ST1-IV strains isolated in Japan are PVL-negative and appear to have a different phylogenetic origin from the conventional USA400 lineage [9,13,14]. Consequently, Japanese guidelines now define PVL-negative ST1-IV strains as the USA400/J clone, distinguishing them from the conventional USA400 clone [5]. The USA400/J clone has increasingly been associated with clinical infections and outbreaks in acute care hospitals and long-term care facilities in Japan [9,[15], [16], [17]]. These observations suggest that ST1-IV strains, traditionally associated with CA-MRSA, have become established in healthcare settings. Understanding the current epidemiologic status of MRSA is, therefore, essential for implementing effective infection control measures. In this study, we conducted an 8-year molecular epidemiologic analysis of MRSA isolates obtained from a single Japanese university hospital in Tokyo to characterize longitudinal changes in circulating MRSA clones and their antimicrobial susceptibility profiles.

## Methods

### Bacterial strains

In total, 1479 MRSA isolates were collected continuously between 2016 and 2023 at the Tokyo Medical University Hachioji Medical Centre, Tokyo, Japan. Duplicate isolates obtained from the same patient were excluded from the analysis. All isolates were newly analyzed in the present study. SCC*mec* typing and virulence gene detection were performed for all 1479 isolates, whereas MLST and PFGE analyses were performed only on the PVL-negative SCC*mec* type IV isolates.

Reference strains representing each SCC*mec* type were used, including NCTC10442 (type I), N315 (type II), 85/2082 (type III), JCSC4469 (type IV), and WIS (type V) [18]. The MRSA N315 strain and the methicillin-susceptible *S. aureus* JCM2874 strain were used as reference strains for antimicrobial susceptibility testing, whereas the USA300 clone JCSC6774 strain and the USA400 clone JCSC6745 strain served as reference strains for PFGE.

### Detection of S. aureus and MRSA

The specimens were spread onto mannitol salt agar (BD Biosciences) and incubated at 37°C for 48 hours. After the proliferating colonies were isolated, Gram staining was performed using Faber G (NISSUI). Strains confirmed as Gram-positive cocci and tested positive in the coagulase test with PS Latex (Eiken Chemical) were identified as *S. aureus*. Strains in which *mecA* was detected via polymerase chain reaction (PCR) were classified as MRSA.

### PCR, SCCmec typing, MLST, and PFGE analysis

PCR assays were performed to confirm the presence of the PVL gene (*lukS/F-PV*) and the TSST-1 gene (*tst*) [18]. SCC*mec* typing and MLST were conducted as previously described [18]. Strains initially identified as SCC*mec* type I by conventional PCR were re-analyzed using primers reported by Takadama et al*.* [19], owing to a 12 bp deletion in the *ccrB2* gene of SCC*mec*. The nucleotide sequences of housekeeping genes were determined using an ABI 3500 Genetic Analyzer (Thermo Fisher Scientific). PFGE patterns were generated through digestion with SmaI and analyzed using BioNumerics version 7.1 (Applied Maths). A dendrogram based on the PFGE banding patterns was constructed using the unweighted pair group method with an arithmetic mean based on the Dice coefficient (band tolerance, 1.5%; optimization, 1.5%). Groups showing 80% or greater similarity in PFGE banding patterns were classified as the same clone. Strains that were SCC*mec* type IV and ST1, were PVL-negative, and exhibited 80% or greater similarity to the USA400 reference strain (JCSC6745) in PFGE banding patterns were defined as USA400/J clones. Thus, USA400/J was distinguished from the original USA400 lineage by its PVL-negative status, despite showing a PFGE banding pattern similar to that of strain JCSC6745. Furthermore, PVL-negative ST8 strains carrying SCC*mec* type IVl were defined as ST8 CA-MRSA/J (CA-MRSA/J) clones, as described by Iwao et al. [20]. SCC*mec* type IVl was confirmed by PCR using the primer sets reported by Iwao et al. [20].

### Antimicrobial susceptibility

Antimicrobial susceptibility was evaluated by determining the minimum inhibitory concentration (MIC) [21]. The MIC was determined using the agar dilution method in accordance with the Clinical and Laboratory Standards Institute guidelines. The antimicrobial agents tested included oxacillin (Tokyo Chemical Industry), levofloxacin (Tokyo Chemical Industry), clarithromycin (Tokyo Chemical Industry), clindamycin (Tokyo Chemical Industry), gentamicin (FUJIFILM Wako Pure Chemical), minocycline (Tokyo Chemical Industry), vancomycin (FUJIFILM Wako Pure Chemical), teicoplanin (Abcam), and linezolid (BLD Pharm). Resistance break points for each agent were set according to the Clinical and Laboratory Standards Institute guidelines [22].

### Statistical analysis

Detection rates and antimicrobial resistance rates were compared using the chi-square test, with *P* < 0.05 considered statistically significant.

## Results

### Characteristics of MRSA isolates

In total, 1479 non-duplicate MRSA isolates were collected during the 8-year study period (Table 1). Respiratory tract specimens were the most common source throughout the study period, accounting for 35.9%-52.6% of isolates annually. Although the proportions of isolates obtained from pus and wound/skin specimens varied among the individual years, no consistent increasing trend was observed during the study period.

**Table 1:** Clinical characteristics of MRSA isolates according to year of isolation.

Category	Year of isolation (%)								Total (n = 1479)
	2016 (n = 196)	2017 (n = 241)	2018 (n = 172)	2019 (n = 195)	2020 (n = 149)	2021 (n = 232)	2022 (n = 154)	2023 (n = 140)	
**Patient status**									
Inpatient	135 (68.9)	141 (58.5)	108 (62.8)	105 (53.8)	86 (57.7)	114 (49.1)	64 (41.6)	58 (41.4)	811 (54.8)
Outpatient	0 (0.0)	0 (0.0)	64 (37.2)	60 (30.8)	35 (23.5)	0 (0.0)	0 (0.0)	0 (0.0)	159 (10.8)
No data	61 (31.1)	100 (41.5)	0 (0.0)	30 (15.4)	28 (18.8)	118 (50.9)	90 (58.4)	82 (58.6)	509 (34.4)
**Clinical department**									
Breast surgery	1 (0.5)	0 (0.0)	1 (0.6)	2 (1.0)	0 (0.0)	0 (0.0)	1 (0.6)	1 (0.7)	6 (0.4)
Cardiology	11 (5.6)	3 (1.2)	7 (4.1)	11 (5.6)	9 (6.0)	8 (3.4)	7 (4.5)	6 (4.3)	62 (4.2)
Cardiovascular surgery	7 (3.6)	4 (1.7)	13 (7.6)	4 (2.1)	4 (2.7)	9 (3.9)	8 (5.2)	1 (0.7)	50 (3.4)
Dermatology	14 (7.1)	30 (12.4)	8 (4.7)	20 (10.3)	9 (6.0)	15 (6.5)	11 (7.1)	15 (10.7)	122 (8.2)
Emergency medicine	37 (18.9)	58 (24.1)	44 (25.6)	39 (20.0)	51 (34.2)	73 (31.5)	58 (37.7)	43 (30.7)	403 (27.2)
Endocrinology	0 (0.0)	0 (0.0)	3 (1.7)	2 (1.0)	2 (1.3)	1 (0.4)	1 (0.6)	0 (0.0)	9 (0.6)
Gastroenterology	1 (0.5)	3 (1.2)	6 (3.5)	4 (2.1)	4 (2.7)	5 (2.2)	5 (3.2)	5 (3.6)	33 (2.2)
Gastrointestinal surgery	9 (4.6)	16 (6.6)	6 (3.5)	9 (4.6)	6 (4.0)	4 (1.7)	4 (2.6)	2 (1.4)	56 (3.8)
General medicine	0 (0.0)	0 (0.0)	3 (1.7)	1 (0.5)	1 (0.7)	0 (0.0)	1 (0.6)	0 (0.0)	6 (0.4)
Geriatrics	2 (1.0)	3 (1.2)	0 (0.0)	0 (0.0)	0 (0.0)	0 (0.0)	2 (1.3)	1 (0.7)	8 (0.5)
Hematology	0 (0.0)	0 (0.0)	0 (0.0)	0 (0.0)	1 (0.7)	0 (0.0)	0 (0.0)	0 (0.0)	1 (0.1)
Infectious diseases	0 (0.0)	2 (0.8)	0 (0.0)	0 (0.0)	5 (3.4)	14 (6.0)	2 (1.3)	1 (0.7)	24 (1.6)
Intensive care unit	3 (1.5)	4 (1.7)	1 (0.6)	3 (1.5)	3 (2.0)	1 (0.4)	0 (0.0)	0 (0.0)	15 (1.0)
Medical oncology	0 (0.0)	2 (0.8)	1 (0.6)	0 (0.0)	0 (0.0)	0 (0.0)	0 (0.0)	0 (0.0)	3 (0.2)
Nephrology	19 (9.7)	28 (11.6)	12 (7.0)	11 (5.6)	8 (5.4)	11 (4.7)	9 (5.8)	6 (4.3)	104 (7.0)
Neurology	5 (2.6)	6 (2.5)	2 (1.2)	3 (1.5)	5 (3.4)	8 (3.4)	4 (2.6)	5 (3.6)	38 (2.6)
Neurosurgery	7 (3.6)	3 (1.2)	9 (5.2)	6 (3.1)	5 (3.4)	5 (2.2)	4 (2.6)	5 (3.6)	44 (3.0)
Obstetrics and gynecology	3 (1.5)	2 (0.8)	2 (1.2)	0 (0.0)	2 (1.3)	2 (0.9)	3 (1.9)	7 (5.0)	21 (1.4)
Ophthalmology	3 (1.5)	1 (0.4)	0 (0.0)	0 (0.0)	1 (0.7)	1 (0.4)	1 (0.6)	3 (2.1)	10 (0.7)
Oral and maxillofacial Surgery	0 (0.0)	1 (0.4)	0 (0.0)	1 (0.5)	0 (0.0)	1 (0.4)	0 (0.0)	2 (1.4)	5 (0.3)
Orthopedic surgery	2 (1.0)	2 (0.8)	5 (2.9)	3 (1.5)	2 (1.3)	3 (1.3)	2 (1.3)	1 (0.7)	20 (1.4)
Otolaryngology	20 (10.2)	13 (5.4)	13 (7.6)	4 (2.1)	4 (2.7)	9 (3.9)	5 (3.2)	10 (7.1)	78 (5.3)
Pediatrics	5 (2.6)	6 (2.5)	4 (2.3)	10 (5.1)	4 (2.7)	3 (1.3)	1 (0.6)	2 (1.4)	35 (2.4)
Pulmonology	8 (4.1)	3 (1.2)	3 (1.7)	2 (1.0)	3 (2.0)	4 (1.7)	4 (2.6)	6 (4.3)	33 (2.2)
Radiology	0 (0.0)	0 (0.0)	0 (0.0)	0 (0.0)	0 (0.0)	0 (0.0)	1 (0.6)	0 (0.0)	1 (0.1)
Reconstructive surgery	16 (8.2)	27 (11.2)	17 (9.9)	22 (11.3)	10 (6.7)	18 (7.8)	6 (3.9)	6 (4.3)	122 (8.2)
Rheumatology	3 (1.5)	0 (0.0)	0 (0.0)	1 (0.5)	1 (0.7)	0 (0.0)	1 (0.6)	0 (0.0)	6 (0.4)
Transplant surgery	7 (3.6)	4 (1.7)	3 (1.7)	3 (1.5)	4 (2.7)	6 (2.6)	4 (2.6)	5 (3.6)	36 (2.4)
Urology	13 (6.6)	14 (5.8)	8 (4.7)	17 (8.7)	4 (2.7)	6 (2.6)	8 (5.2)	7 (5.0)	77 (5.2)
No data	0 (0.0)	6 (2.5)	1 (0.6)	17 (8.7)	1 (0.7)	25 (10.8)	1 (0.6)	0 (0.0)	51 (3.4)
**Specimen source**									
Blood	7 (3.6)	14 (5.8)	11 (6.4)	12 (6.2)	7 (4.7)	7 (3.0)	8 (5.2)	15 (10.7)	81 (5.5)
Body fluids	0 (0.0)	4 (1.7)	3 (1.7)	2 (1.0)	2 (1.3)	1 (0.4)	0 (0.0)	0 (0.0)	12 (0.8)
Catheter/device	7 (3.6)	4 (1.7)	1 (0.6)	4 (2.1)	2 (1.3)	1 (0.4)	3 (1.9)	0 (0.0)	22 (1.5)
Ear/sinus	7 (3.6)	7 (2.9)	7 (4.1)	4 (2.1)	3 (2.0)	2 (0.9)	2 (1.3)	5 (3.6)	37 (2.5)
Environmental	0 (0.0)	0 (0.0)	0 (0.0)	5 (2.6)	0 (0.0)	0 (0.0)	0 (0.0)	0 (0.0)	5 (0.3)
Eye	2 (1.0)	1 (0.4)	0 (0.0)	0 (0.0)	0 (0.0)	1 (0.4)	1 (0.6)	3 (2.1)	8 (0.5)
Genital tract	3 (1.5)	1 (0.4)	0 (0.0)	1 (0.5)	1 (0.7)	1 (0.4)	2 (1.3)	6 (4.3)	15 (1.0)
Nasal-oropharyngeal	8 (4.1)	15 (6.2)	5 (2.9)	4 (2.1)	2 (1.3)	2 (0.9)	1 (0.6)	7 (5.0)	44 (3.0)
Pus	13 (6.6)	23 (9.5)	17 (9.9)	21 (10.8)	17 (11.4)	28 (12.1)	20 (13.0)	13 (9.3)	152 (10.3)
Respiratory tract	87 (44.4)	90 (37.3)	82 (47.7)	70 (35.9)	76 (51.0)	122 (52.6)	73 (47.4)	60 (42.9)	660 (44.6)
Stool	7 (3.6)	4 (1.7)	4 (2.3)	2 (1.0)	4 (2.7)	4 (1.7)	0 (0.0)	3 (2.1)	28 (1.9)
Urine	22 (11.2)	25 (10.4)	20 (11.6)	28 (14.4)	19 (12.8)	16 (6.9)	24 (15.6)	16 (11.4)	170 (11.5)
Wound/skin	32 (16.3)	42 (17.4)	21 (12.2)	28 (14.4)	11 (7.4)	21 (9.1)	19 (12.3)	12 (8.6)	186 (12.6)
No data	1 (0.5)	11 (4.6)	1 (0.6)	14 (7.2)	5 (3.4)	26 (11.2)	1 (0.6)	0 (0.0)	59 (4.0)

Abbreviation: MRSA, methicillin-resistant *Staphylococcus aureus.*

### Annual transition of SCCmec types

SCC*mec* typing was performed to identify the predominant MRSA strains (Figure 1). The proportion of SCC*mec* type II strains, corresponding to HA-MRSA, decreased significantly from 50.5% in 2016 to 12.1% in 2023 (*P* < 0.01), whereas the proportion of type IV strains, corresponding to CA-MRSA, increased significantly from 45.9% to 86.4% over the same period (*P* < 0.01).

**Figure 1:**
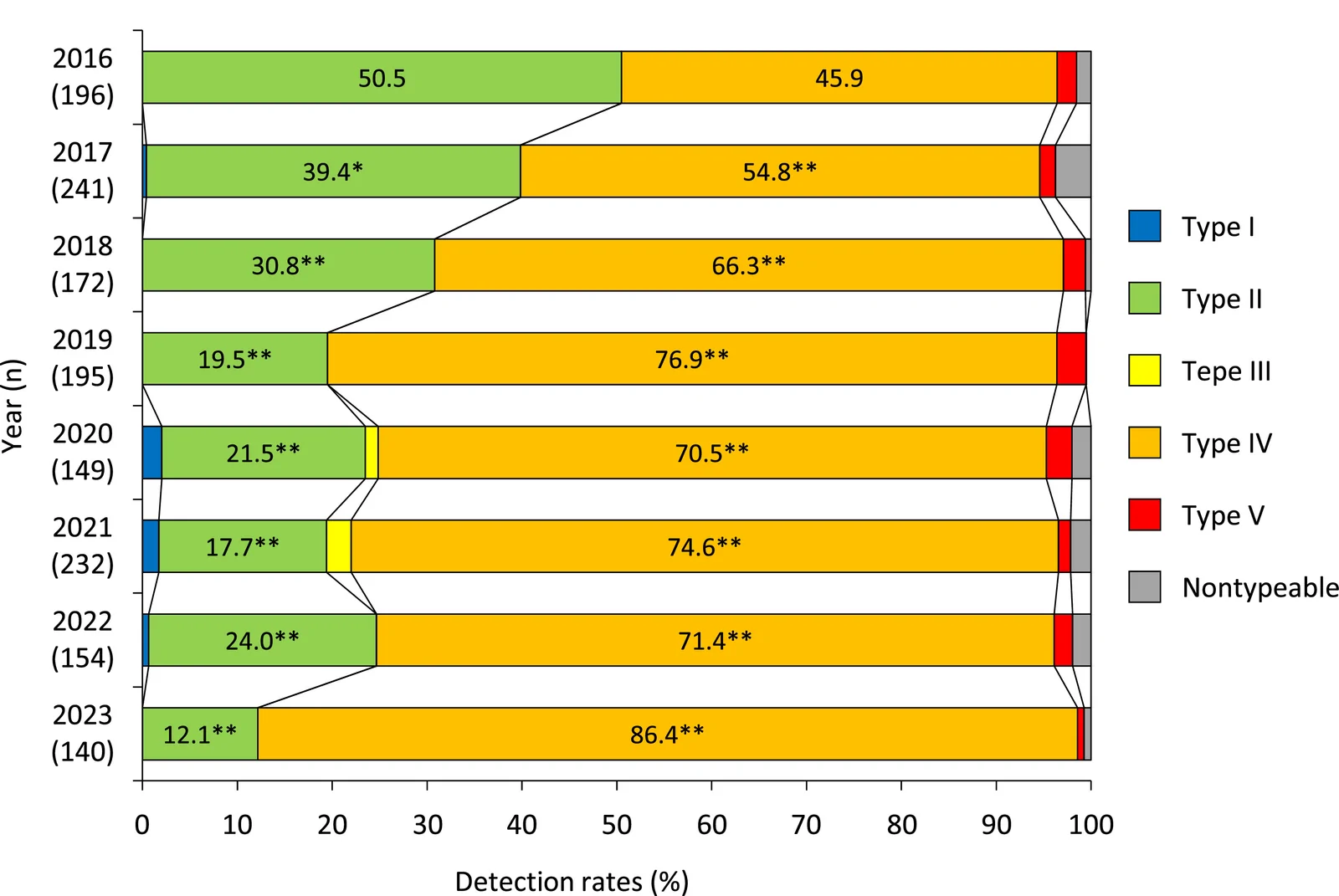
Annual transition of the detection rates of staphylococcal cassette chromosome *mec* (SCC*mec*) types among methicillin-resistant *Staphylococcus aureus* (MRSA) isolates from 2016 to 2023. This figure illustrates the annual changes in the distribution of SCC*mec* types among the MRSA isolates collected between 2016 and 2023. Statistical comparisons were performed using the chi-square test, comparing each year with 2016. Asterisks (*) indicate statistically significant differences (*P < 0.05 and **P < 0.01 vs 2016).

### Toxin gene carrying status

The prevalence of toxin genes in MRSA was assessed by evaluating the proportion of isolates positive for TSST-1, a toxin characteristic of HA-MRSA, and PVL, a toxin characteristic of CA-MRSA (Figure 2). The PVL positivity rate ranged from 6.4% to 15.9%, with little variation during the study period, indicating that PVL-negative MRSA remained the predominant type in the hospital.

**Figure 2:**
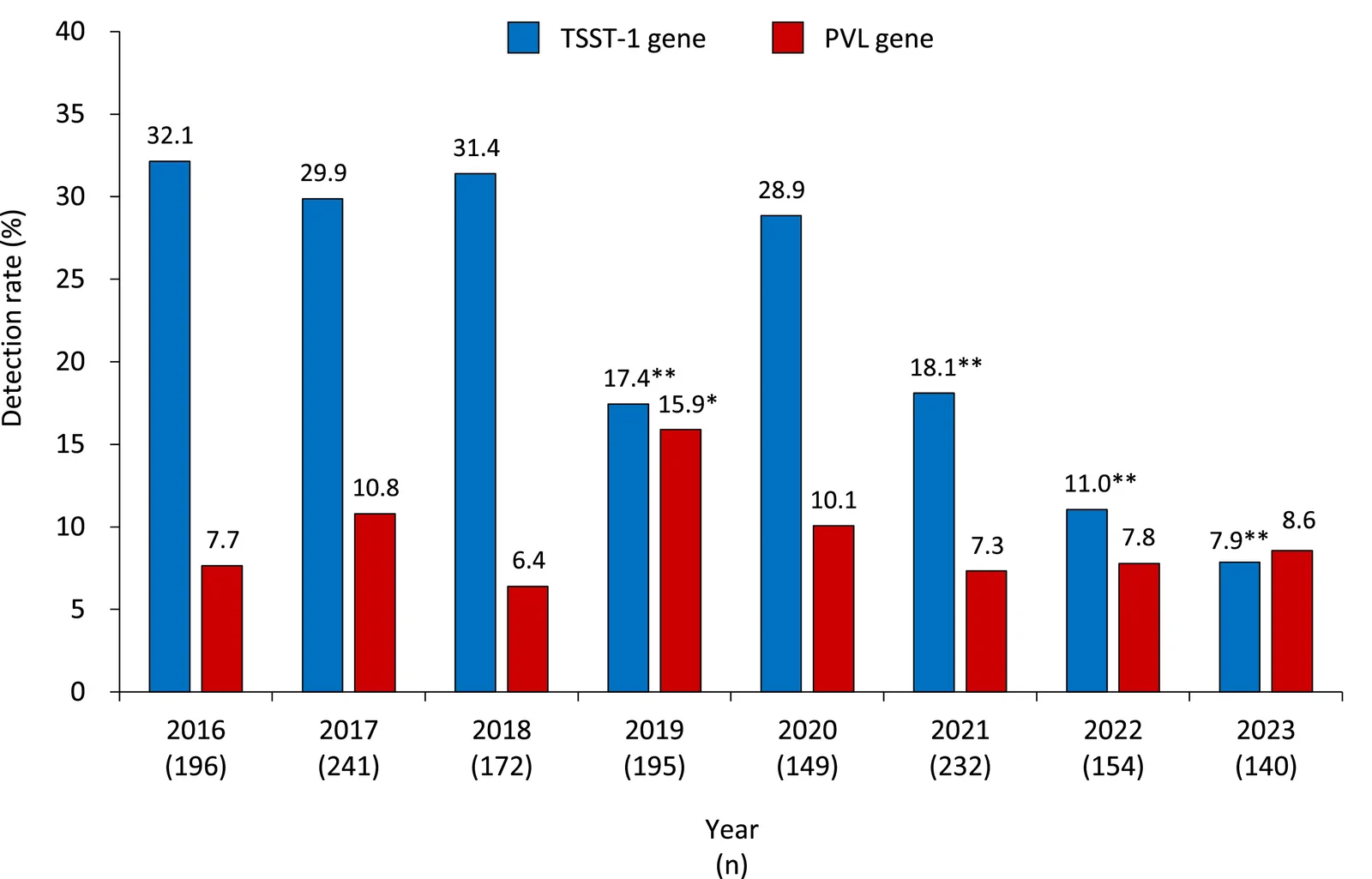
Annual transition of the detection rates of TSST-1 and PVL genes in methicillin-resistant *Staphylococcus aureus* (MRSA) isolates from 2016 to 2023. This figure illustrates the annual changes in the detection rates of the TSST-1 gene (*tst*) and the PVL gene (*lukS/F-PV*) among MRSA isolates collected between 2016 and 2023. Statistical comparisons were performed using the chi-square test, comparing each year with 2016. Asterisks (*) indicate statistically significant differences (*P < 0.05 and **P < 0.01 vs 2016). PVL, Panton–Valentine leukocidin; TSST-1, toxic shock syndrome toxin-1.

### Annual transition of predominant MRSA clones

Molecular epidemiologic analyses using MLST and PFGE were performed on PVL-negative SCC*mec* type IV strains, which were the strains most frequently isolated in the hospital. MLST revealed that CC1 strains accounted for 61.3%-71.6% of the strains, whereas CC8 strains accounted for 19.0%-33.3%, indicating that CC1 and CC8 were the major genotypes (Supplementary Figure 1). Clustering based on the similarity of PFGE banding patterns showed that the USA400/J clone was predominant among the CC1 strains, whereas the CA-MRSA/J clone was predominant among the CC8 strains (Figure 3). During the study period, the USA400/J clone accounted for 28.0%-54.1% of the isolates, whereas the CA-MRSA/J clone accounted for 6.4%-25.3% (Supplementary Figure 2). These findings indicate that the USA400/J clone was the predominant epidemic strain in the hospital.

**Figure 3:**
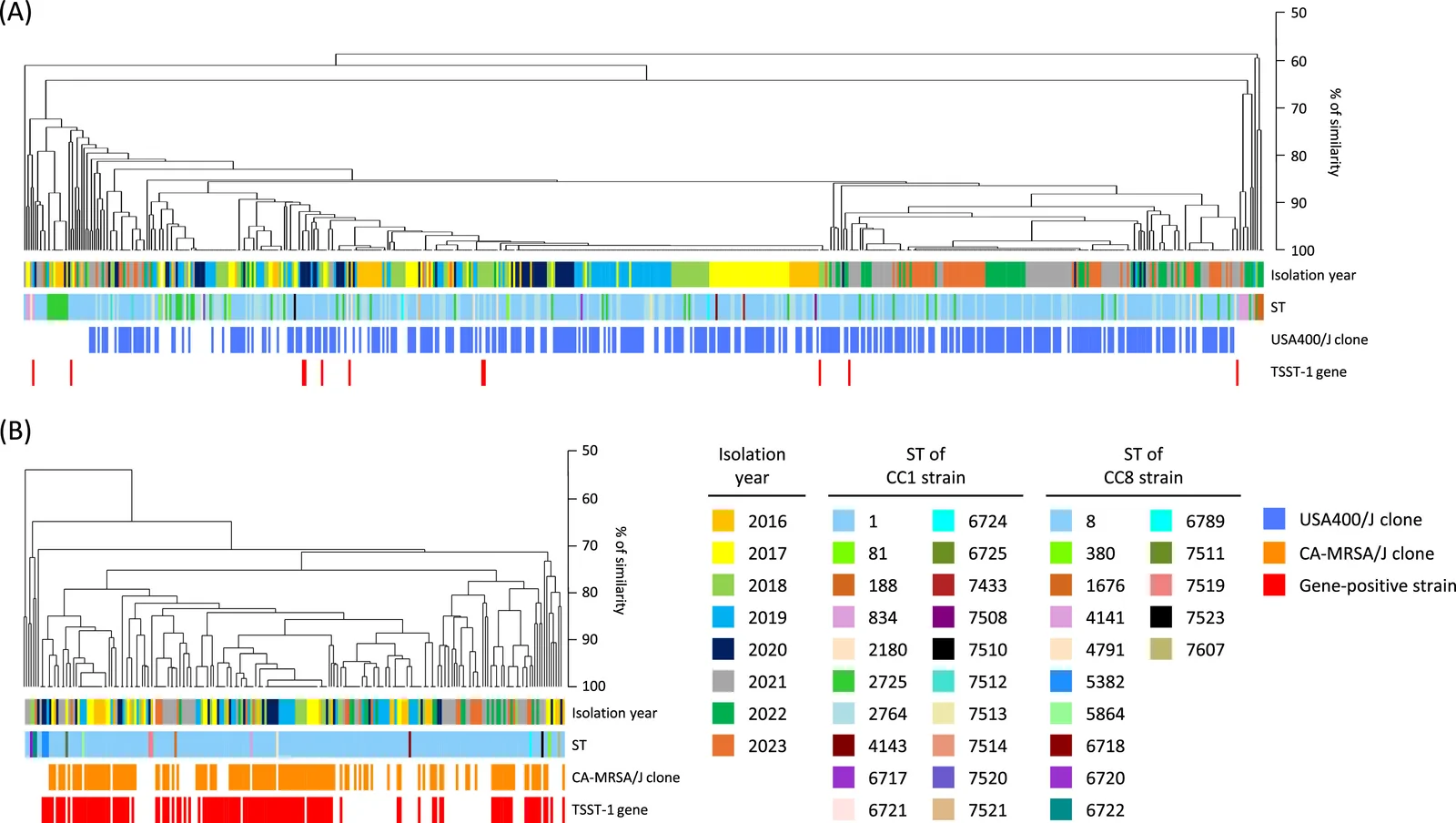
Pulsed-field gel electrophoresis (PFGE) analysis of PVL-negative methicillin-resistant *Staphylococcus aureus* (MRSA) SCC*mec* type IV isolates obtained from the hospital. (a) PFGE profiles of CC1-IV strains. PVL-negative ST1-IV isolates exhibiting high similarity to the reference USA400 clone (JCSC6745) were classified as USA400/J clones. (b) PFGE profiles of CC8-IV strains. Predominant isolates carrying SCC*mec* type IVl were classified as ST8 CA-MRSA/J (CA-MRSA/J) clones. CA-MRSA, community-associated methicillin-resistant *Staphylococcus aureus*; CC, clonal complex; PVL, Panton–Valentine leukocidin; SCC*mec*, staphylococcal cassette chromosome *mec*; ST, sequence type.

### Antimicrobial susceptibility of predominant MRSA clones

For the antimicrobial susceptibility test, the predominant PFGE types within the USA400/J and CA-MRSA/J clones were selected based on PFGE analysis. The antimicrobial susceptibilities of the USA400/J and CA-MRSA/J clones were compared (Table 2). Both clones were highly susceptible to the anti-MRSA agents (vancomycin, teicoplanin, and linezolid). The resistance rates to the quinolone-class agent levofloxacin and the macrolide-class agent clarithromycin were significantly higher in the USA400/J clone than in the CA-MRSA/J clone (*P* < 0.01), whereas the resistance rate to the aminoglycoside-class agent gentamicin was significantly higher in the CA-MRSA/J clone (*P* < 0.01). The resistance rates to the lincosamide-class agent clindamycin and the tetracycline-class agent minocycline were also significantly higher in the CA-MRSA/J clone than in the USA400/J clone (*P* < 0.05).

**Table 2:** Annual transition of antimicrobial susceptibilities in USA400/J clone and ST8 CA-MRSA/J (CA-MRSA/J) clone isolated in this study.

Antimicrobial agent	USA400/J clone (n = 352)		CA-MRSA/J clone (n = 77)		*P*-value
	MIC_50_/MIC_90_ (µg/mL)	R (%)	MIC_50_/MIC_90_ (µg/mL)	R (%)	
Oxacillin	32/64	98.9	32/64	97.4	0.32
Levofloxacin	32/128	100.0	0.25/8	16.9	<0.01
Clarithromycin	4/≥256	40.6	0.125/256	18.2	<0.01
Clindamycin	≤0.063/≤0.063	2.3	≤0.063/0.125	7.8	<0.05
Gentamicin	0.125/16	10.2	32/64	79.2	<0.01
Minocycline	≤0.063/0.125	0.6	≤0.063/0.25	3.9	<0.05
Vancomycin	1/1	0.0	1/1	0.0	-
Teicoplanin	1/1	0.0	1/2	0.0	-
Linezolid	1/2	0.0	1/2	0.0	-

MIC_50_/MIC_90_ values indicate the MICs (µg/mL) that inhibit the growth of 50% and 90% of strains, respectively.

Abbreviations: MIC, minimum inhibitory concentration; R (%), rate of resistant strains; ST, sequence type.

Among the tested agents, levofloxacin exhibited the most pronounced difference in resistance between the two clones. MIC distributions were analyzed to compare susceptibilities (Figure 4). In the USA400/J clone, 98.9% of the isolates were associated with MIC values ≥16 µg/mL, indicating intermediate-to-high–level resistance. In contrast, 83.1% of CA-MRSA/J clone isolates were associated with MIC values of 0.063-1 µg/mL, indicating higher susceptibility than the USA400/J clone.

**Figure 4:**
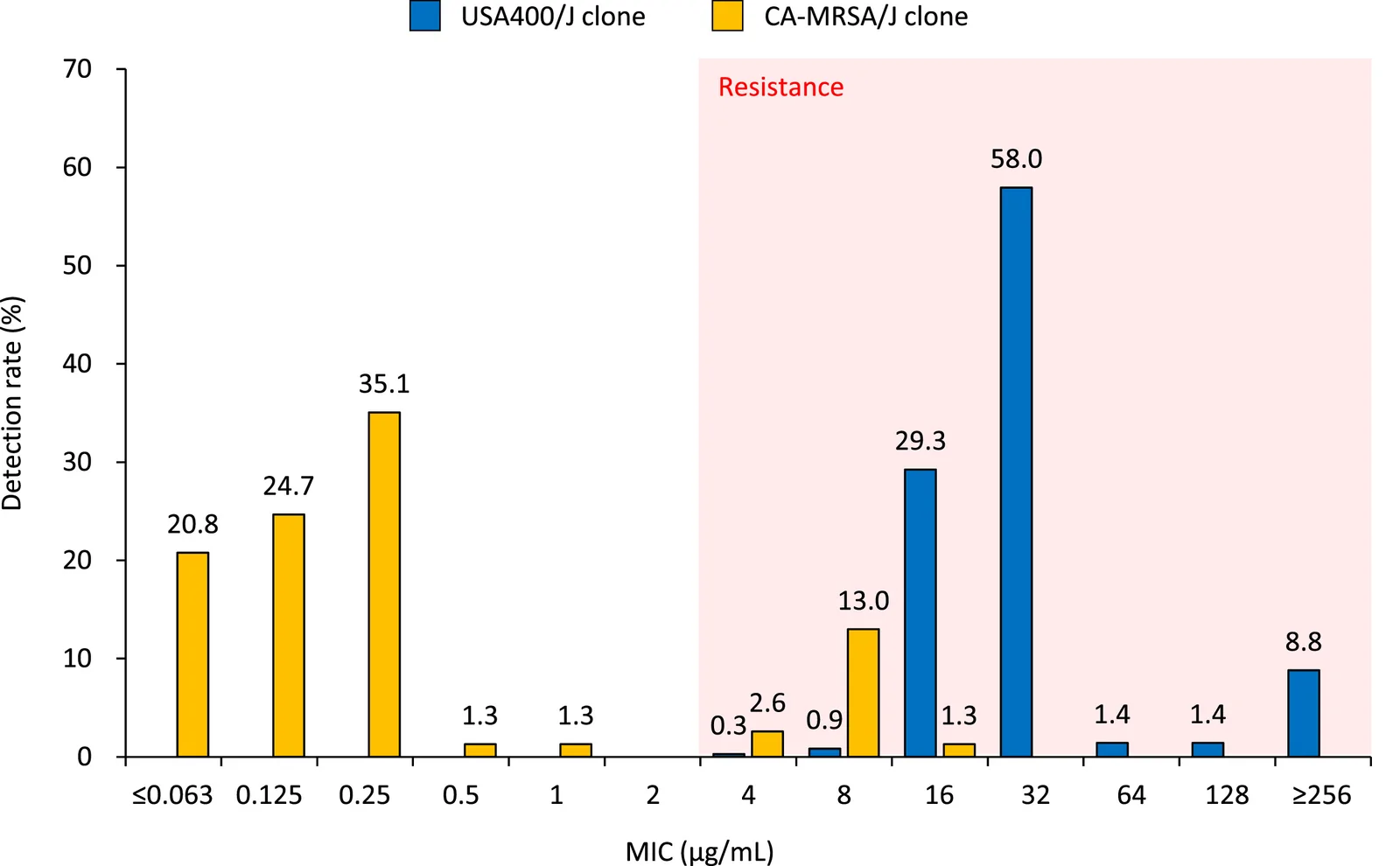
Comparison of the levofloxacin minimum inhibitor concentration (MIC) distribution between USA400/J and ST8 CA-MRSA/J (CA-MRSA/J) clones isolated in this study. Levofloxacin MICs were determined for the clinical isolates comprising the USA400/J and CA-MRSA/J clones using the agar dilution method. The distribution is presented as the percentage of isolates associated with each MIC value. According to the criteria used in this study, isolates associated with MIC values ≥4 µg/mL were defined as resistant. CA-MRSA, community-associated methicillin-resistant *Staphylococcus aureus*; MRSA, methicillin-resistant *Staphylococcus aureus*.

## Discussion

In this 8-year molecular epidemiologic study, we observed a shift in the predominant SCC*mec* type from type II to type IV, with the PVL-negative ST1-IV USA400/J clone becoming predominant. In a previous study conducted in the same region between 2010 and 2014, SCC*mec* type IV strains accounted for 12.4%-45.3% of MRSA isolates [9]. Together with our findings, this suggests a long-term shift toward SCC*mec* type IV MRSA in this hospital. Although similar increases in SCC*mec* type IV MRSA have been reported worldwide, the factors driving this shift remain unclear [23,24].

The predominance of USA400/J may partly explain the observed increase in SCC*mec* type IV MRSA. In contrast to conventional USA400, USA400/J is PVL-negative and has been increasingly associated with health care settings in Japan. Its expansion despite the absence of PVL suggests that factors other than PVL may contribute to its persistence and transmission in health care settings. PVL-negative MRSA strains exhibiting characteristics similar to those of the USA400 clone have also been reported in Australia, Brazil, and Europe [25,26]. Previously, we conducted phylogenetic analyses based on genome sequencing of ST1-IV isolates obtained from Japan and overseas [18]. These analyses revealed that USA400/J is distributed across multiple hospitals in Japan, indicating that it is not restricted to a single institution. This distribution suggests that USA400/J may possess characteristics that facilitate its persistence and transmission in healthcare settings. Yamazaki et al. [17] reported that strains with characteristics of USA400/J can regulate the accessory gene regulator via cytosine methylation, facilitating adaptation to hospital settings. Such a regulatory mechanism may partly contribute to the persistence of this clone in health care settings.

The CA-MRSA/J clone identified in this study was the second-most prevalent strain after the USA400/J clone. This clone is similar to the USA300 clone, which was first isolated in Japan in 2003 [27]. It has also been reported to exhibit enhanced surface structures that facilitate adhesion to and invasion of human cells compared with other clones [27]. Consequently, these characteristics may underlie its higher invasiveness, and continued surveillance is necessary.

A comparison of the antimicrobial susceptibility profiles of the USA400/J and CA-MRSA/J clones showed that the USA400/J clone exhibited intermediate-to-high–level resistance to levofloxacin. The typical USA400 clone has previously been reported to be susceptible to most antimicrobial agents other than β-lactams [28]. However, in the present study, isolates resistant to multiple antimicrobial agents, including levofloxacin, were identified. Notable differences in antimicrobial susceptibility were observed between the two clones, with a pronounced disparity in susceptibility to levofloxacin. These findings suggest that levofloxacin susceptibility may serve as a practical phenotypic marker for predicting the genotypes of SCC*mec* type IV strains.

This study has several notable limitations. Detailed clinical information on the patients, including age, sex, diagnosis, symptoms, and circumstances of specimen collection, was lacking for the MRSA isolates. Although information on inpatient and outpatient statuses was available, the interval between hospital admission and isolate collection was not available for inpatients. In addition, there was no sufficient clinical information to reliably distinguish infection from colonization. Therefore, isolates from inpatients could not be classified according to the timing of isolation after admission, and we were unable to reliably distinguish between community-onset and health care–associated cases. These limitations should be considered when interpreting the epidemiologic significance of SCC*mec* type IV MRSA in this study. Furthermore, because detailed patient-level epidemiologic and transmission data were unavailable, we could not determine whether the observed increase in the USA400/J clone reflected a broader epidemiologic shift or the prolonged transmission of this clone within the hospital, including possible outbreak-related clustering. In addition, molecular epidemiologic analyses were performed using MLST and PFGE. Although these methods are useful for epidemiologic surveillance, they have limited discriminatory power and cannot fully resolve the genetic relatedness among closely related isolates. Whole genome sequencing would provide a higher-resolution assessment of clonal relationships and transmission dynamics and enable more detailed comparisons with related strains from other institutions in Japan and abroad. In addition, because this study was conducted at a single hospital, the results may not adequately reflect the regional variations or diversity of circulating strains across Japan. These limitations may have influenced the interpretation of our findings.

## Conclusions

The findings of this study provide valuable guidance for antimicrobial selection in clinical practice and may inform the management of infections for which early therapy initiation is critical. Continued surveillance of the USA400/J clone and other prevalent clones, together with monitoring changes in antimicrobial susceptibility, is warranted.

## Funding

This work was supported by a 10.13039/501100001691Japan Society for the Promotion of Science Grant-in-Aid for Scientific Research (C) (grant numbers 18K06797 and 23K07928) and Uehara Memorial Foundation Research Grant for Infection and Chemotherapy to H.N.

## Ethical approval

The study protocol was approved by the Ethics Committee of Tokyo University of Pharmacy and Life Sciences (approval number W-2024-010). Informed consent was waived because the data and isolates were collected without any direct clinical interaction with the patients.

## CRediT authorship contribution statement

**Takuma Yoshida:** Writing – original draft, Visualization, Methodology, Investigation, Formal analysis, Data curation, Conceptualization. **Rika Sawara:** Investigation, Formal analysis, Data curation. **Yuma Tomida:** Investigation, Formal analysis, Data curation. **Mayu Sato:** Investigation, Formal analysis, Data curation. **Yuki Okura:** Investigation, Formal analysis, Data curation. **Hidetoshi Masuda:** Investigation, Formal analysis, Data curation. **Taiga Natori:** Investigation, Formal analysis, Data curation. **Takemi Ozeki:** Methodology, Investigation, Formal analysis, Data curation. **Tatsuya Numadate:** Investigation, Formal analysis, Data curation. **Natsuki Oda:** Investigation, Formal analysis, Data curation. **Megumi Sakaihori:** Investigation, Formal analysis, Data curation. **Rika Watanabe:** Investigation, Formal analysis, Data curation. **Hinako Kawasaki:** Investigation, Formal analysis, Data curation. **Natsuki Fukuda:** Investigation, Formal analysis, Data curation. **Tomomi Akasu:** Investigation, Formal analysis, Data curation. **Shunsuke Takadama:** Investigation, Formal analysis, Data curation. **Hiroshi Kaneko:** Validation, Methodology, Investigation, Formal analysis, Data curation. **Norihisa Noguchi:** Validation, Supervision, Project administration, Conceptualization. **Yuji Hirai:** Validation, Resources. **Hidemasa Nakaminami:** Writing – review & editing, Visualization, Validation, Supervision, Project administration, Methodology, Funding acquisition, Conceptualization.

## Declaration of competing interest

The authors have no competing interests to declare.

## Appendix Supplementary materials

Image, application 1

Image, application 2
